# An annotated dataset of Egyptian fruit bat vocalizations across varying contexts and during vocal ontogeny

**DOI:** 10.1038/sdata.2017.143

**Published:** 2017-10-03

**Authors:** Yosef Prat, Mor Taub, Ester Pratt, Yossi Yovel

**Affiliations:** 1 Department of Zoology, Faculty of Life sciences, Tel Aviv University, Tel Aviv, Israel

**Keywords:** Animal behaviour, Databases, Evolution of language

## Abstract

Animal acoustic communication research depends on our ability to record the vocal behaviour of different species. Only rarely do we have the opportunity to continuously follow the vocal behaviour of a group of individuals of the same species for a long period of time. Here, we provide a database of Egyptian fruit bat vocalizations, which were continuously recorded in the lab in several groups simultaneously for more than a year. The dataset includes almost 300,000 files, a few seconds each, containing social vocalizations and representing the complete vocal repertoire used by the bats in the experiment period. Around 90,000 files are annotated with details about the individuals involved in the vocal interactions, their behaviours and the context. Moreover, the data include the complete vocal ontogeny of pups, from birth to adulthood, in different conditions (e.g., isolated or in a group). We hope that this comprehensive database will stimulate studies that will enhance our understanding of bat, and mammal, social vocal communication.

## Background & Summary

**Figure 1:**
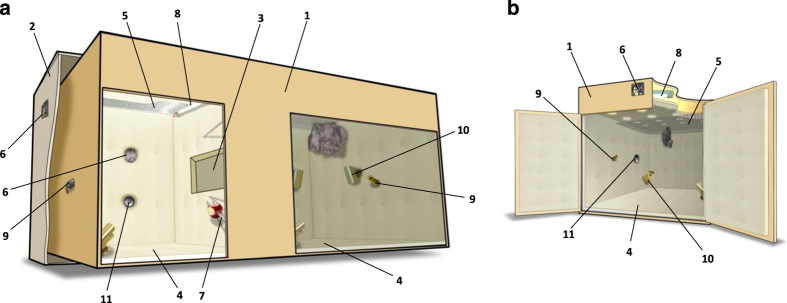
The recording setup. (**a**) *Colony* chamber (length: 190 cm; width: 90 cm; height: 82 cm). Chambers of this type housed *Colony* treatment adults and pups, as well as groups of weaned pups in both treatments. (**b**) *Isolation* chamber (length: 120 cm; width: 70 cm; height: 60 cm). Chambers of this type housed *Isolation* treatment mothers with their pre-weaned pups. Legend: 1. wooden box; 2. outer box for acoustic isolation; 3. A window allowing transition between the ‘roost’ compartment and the ‘foraging’ compartment, and allowing some light from the ‘foraging’ compartment to penetrate the ‘roost’ during the day; 4. foam for echoes reduction; 5. plastic mesh for facilitating hanging from the ceiling; 6. airflow ventilators; 7. feeding skewers; 8. lights (active during daytime); 9. ultrasonic microphones; 10. infra-red sensitive video cameras; 11. loudspeakers (not used in this study).

Comparative research of nonhuman animals can potentially shed light on the evolution of language and speech^1^. For instance, the study of animal vocal communication may reveal the roots of syntax and semantics^2,3^. Nonhuman vocalizations are often cryptic to a human observer, and with little prior knowledge about the animal-relevant acoustics, identifying essential information in them becomes an arduous task. Still, the discovery of nuances, which may be subtle to the human ear but important to the communicating animal, may become plausible if facilitated by large recording datasets. Here we present an extremely large collection of vocalizations of Egyptian fruit bats (*Rousettus aegyptiacus*). Moreover, many of the vocalizations in this database are accompanied by relevant information such as the identities of the emitter and the addressee of the vocalization, and the related behavioural context. Bats are social mammals which use rich vocal communication^4–9^, and have been found to possess the capability of vocal learning^10–13^. Being social animals which almost exclusively interact with each other in the dark, and together with the versatile vocal skills found in this group (e.g., refs 14,15), bats make an interesting model for vocal communication studies. Egyptian fruit bats live in colonies of dozens to thousands, and may live (at least) to the age of 25 years^16^. They are extremely vocal, with most vocal interactions involving mildly-aggressive encounters in the roost^17^, and their vocalizations are composed of sequences of multi-harmonic low-fundamental syllables^13,17^. In this study, all the recordings were conducted in acoustically isolated cages, specifically designed for this purpose (Fig. 1). Bats were housed in these cages for periods of several months and were recorded around the clock with microphones and video cameras, which enabled detailed annotation of the interactions. Importantly, this study did not a-priory focus on specific types of vocalizations (such as songs, alarms, distress, etc.), hence the dataset mostly includes those vocalizations accompanying the everyday pairwise interactions of bats. Furthermore, the data covers the complete repertoire used by the bats during their housing in the experimental setup. The dataset also includes vocalizations of bats which were born inside the experimental setup, and recorded from birth to adulthood, under different experimental conditions (in isolation or in a group). Therefore, this collection enables the tracking of the vocal ontogeny of bat pups^13^. We provide the raw recordings (audio files of a few seconds each), which are usually of high signal-to-noise ratio. Retrieving the relevant, voiced, segments from a recorded audio track is often the first obstacle in analysing audio data. Thus, we provide a fairly accurate segmentation of the data (generated based on the method described in ref. 13), which paves an easy and straightforward way to process and analyse the vocalizations. The presented rich dataset can be potentially used to enhance our understanding of the origins of semantics (as in ref. 17), the ontogeny of a mammalian vocal communication (as in ref. 13), or even the putative use of syntax, as was observed, for instance, in courtship songs of birds^18,19^ and songs of a few bat species^7,20^.

## Methods

### Animal retrieval and care

All adult bats (*Rousettus aegyptiacus*), females in their late pregnancy and males, were caught in a natural roost near Herzliya, Israel. This roost is inhabited by a colony of 5,000 to 10,000 bats. All recorded pups were born in the experimental setup to wild-caught females. The bats were kept in acoustic chambers, large enough to allow flight (Fig. 1), and fed with a variety of fruits. Pups were separated from their mothers, and joined together (if were previously isolated; see below), after all pups were observed feeding on fruit by themselves. All experiments were reviewed and approved by the Institutional Animal Care and Use Committee of Tel Aviv University (Number L-13-016). The use of bats was approved by the Israeli National Park Authority.

### Experimental setup

Two types of chambers were used to house the bats: colony chambers (Fig. 1a) for most of the recordings, and (smaller) isolation chambers (Fig. 1b) for the recording of preweaned isolated pups. The chambers were acoustically isolated (see ref. 13 for isolation verification methods) and their walls were covered with foam to diminish echoes. The chambers were continuously monitored with IR-sensitive cameras and omnidirectional electret ultrasound microphones (Avisoft-Bioacoustics Knowles FG-O). Audio recordings were conducted using Avisoft-Bioacoustics UltraSoundGate 1216H A/D converter with a sampling rate of 250 kHz.

### Recording settings

Two types of treatments are included in our data: *colony* and *isolation* (Table 1). In a *colony* treatment adult bats were housed together, usually a few females and one male, in a colony chamber (Fig. 1a), and pups were born to the females in this chamber. In the *isolation* treatment each pregnant female was housed alone in a private isolation chamber (Fig. 1b), and gave birth to one pup in this chamber. After weaning, pups of both treatments were housed in colony chambers without adults. The recordings were conducted from May 2012 to June 2013, and in February 2014, in chambers of different treatments in parallel, where each chamber was continuously recorded (see Table 1 for recording periods of each group, and Table 2 for treatment assignment of individual bats). Importantly, we include in this database all of the recordings in which our automatic tools identified social calls. Thus, the database can be regarded as practically complete representation of the social vocal communication used by the bats during the recording periods, and statistics about the total usage of vocalizations can be safely drawn. Recordings may only be missing on rare occasions, due to short periods of technical problems, such as power cuts or malfunctioning microphone replacements.

## Data Records

The data (Data Citation 1) consist of:

1. 293,238 recorded audio files (WAV format, sampling rate: 250 kHz, depth: 16 bit). The files are compressed (and can be extracted) using 7-zip (www.7-zip.org).
2. One annotation file: Annotations.csv, with 91,080 annotations. These annotations were obtained from the videos (see below) and include details such as the emitter and context of each vocalization. The content of each column in the annotation file is described in Table 3, and descriptions of contexts and behaviours are depicted in Table 4. Each annotation corresponds to sequences of vocalizations in one file. Most files include a single interaction and, correspondingly, a single annotation, though some files record several interactions (and may be annotated with several annotations). Accordingly, columns 9 and 10 in Annotations.csv specify the location in the file to which each annotation refers (see Table 3).
3. One file describing the audio files: FileInfo.csv, which includes the exact recording time, the recording channel, and the exact time of the voiced segments in each file.
4. One metadata file: Metadata.pdf, with details about the subjects and annotation definitions (Tables 1,2,3,4).
5. A set of audio example files.
6. Example videos of different interactions.
7. A folder with example raw video recordings.
8. A sample Matlab code exemplifying the segmentation and noise-filtering of raw audio recording. A similar process was used for obtaining the start and end positions of voiced segments (given in FileInfo.csv), and for filtering voiceless files; parameter adjustment might be required for specific tasks.

The recorded audio files are divided into folders of no more than 10,000 files for the convenience of use (this division has no significance). Note that two non-annotated files might be different recordings of the same call, if they were recorded at the same time, in the same treatment, in different channels, though it is not necessarily the case, and this is never the case for annotated files. Such duplicates can be excluded by the user by inspecting the recordings themselves. The annotation and metadata files are in comma-separated-value format (CSV) to ease their use with automatic tools, and to allow their direct upload into spreadsheet software. The metadata file includes descriptions of all identifiers in the annotation file. The example files include a few audio files exemplifying different recorded sounds, to facilitate the familiarity of the user with the recorded data. These examples include social call syllables, isolation calls of young pups, echolocation clicks, and examples of background noises (e.g., cage noise, microphone direct hit, etc., which are all rare in this database).

## Technical Validation

The annotation types (contexts or behaviours) were defined by YP and MT. The recordings were annotated using the videos by MT and EP, or by trained students. These observers were certified after annotating a few recording days, which were then validated by an expert (YP or MT). In annotating the recordings we adopted a conservative approach, in which we designated as ‘unknown’ any type of data for which we had any doubt. Despite the training of the observers, some noise might have been introduced during the hundreds of hours of manual annotations, thus we estimated an error rate by a post-hoc quality test: 435 annotated recordings were sampled randomly and were then carefully re-annotated by EP, MT, and YP. Errors were counted when there were either a discrepancy between the post-hoc and the original annotations, or when the post-hoc examination concluded that some doubt still exists. The error rates were 2.1% (95% Confidence-Interval [CI]: 0.8–3.4%) for the emitter identification, 2.1% (95% CI: 0.8–3.4%) for the addressee identification, and 4% (95% CI: 2.2–5.8%) for the context identification. Thus we estimate the accuracy of the annotations as 97.9% for the emitter and addressee, and 96% for the context.

## Usage Notes

The FileInfo.csv file includes automatically generated start and end positions of voiced segments (in samples) for each file. This enables an immediate analysis of the data without the need to apply any pre-processing. However, we encourage users to verify the suitability of the automatic segmentation (given in FileInfo.csv) to their needs by reviewing it in a random sample of recordings. To facilitate such review, and familiarity with the database, we provide a small library of examples of different sounds which might be encountered in the raw recordings, including social calls (which are the core interest of this study, and the most common sounds in the database), echolocation clicks (which are sporadically recorded before or after social calls), cage noises (relatively rare), and pup isolation calls (which are distinct from adults calls). For a usage which is sensitive to possible differences between microphones, one may take advantage of the *Recording channel* (different channels represent different microphones) field in FileInfo.csv (note that some microphones might have been replaced during the experiment, although these replacements were rare).

## Additional Information

**How to cite this article:** Prat, Y. *et al.* An annotated dataset of Egyptian fruit bat vocalizations across varying contexts and during vocal ontogeny. *Sci. Data* 4:170143 doi: 10.1038/sdata.2017.143 (2017).

**Publisher’s note:** Springer Nature remains neutral with regard to jurisdictional claims in published maps and institutional affiliations.

## Supplementary Material



## Figures and Tables

**Table 1:** Recording settings.

**Treatment (ID)**	**Treatment (Type)**	**From Date (yyyy-mm-dd)**	**To Date (yyyy-mm-dd)**	**Social Composition (Bat IDs)**	**Rec. Channel**
1	Colony*	2012-06-01	2012-09-06	101,107,108,112,113,114,115,118	4,11
2	Colony*	2012-06-01	2012-09-05	102,109,110,111,116,119,120	9,12
3	Isolation	2012-07-24	2012-08-26	103,104	2
4	Isolation	2012-07-28	2012-08-26	105,106	3
5	Colony† ‡	2012-08-26	2012-12-12	108,115	7
6	Colony† ‡	2012-08-26	2012-09-20	111,120	10
7	Colony† ‡	2012-08-26	2012-09-20	103,105	5
8	Colony†	2012-12-14	2013-01-02	103,105,108,115	7
9	Colony*	2012-10-01	2012-12-15	111,120,213,214,216,221,226,228,233	3,4
10	Colony*	2012-10-01	2012-12-15	201,202,203,204,205,208,211,218,222,223,225,231	1,2
11	Isolation	2012-10-01	2012-12-17	207,209	11
12	Isolation	2012-11-01	2012-12-17	210,212	9
13	Isolation	2012-10-01	2012-10-31	215,217	9/10§
14	Isolation	2012-10-01	2012-12-17	220,224	6
15	Isolation	2012-10-01	2012-12-17	230,232	12
16	Colony†	2012-12-18	2013-05-12	208,211,216,221,231	5,9/8||
17	Colony†	2012-12-18	2013-05-12	207,210,215,220,230	1,2
18	Colony†	2013-05-13	2013-06-21	207,210,216,221,230	5,8
19	Colony†	2013-05-13	2013-06-21	208,211,215,220,231	1,2
20	Colony†	2014-02-06	2014-02-19	207,208,211,215,216,221	1,2
Colony treatment involves a group of bats housed in a *Colony* chamber (Fig. 1a). Isolation treatment involves a mother and her pup housed in an *Isolation* chamber (Fig. 1a). When a bat ID appears in more than one treatment it was moved from one treatment to the next, always in the order of their start time, and usually at the beginning of the next treatment (exact times of recordings are included in Data Citation 1).					

*The colony includes adults which were captured in the wild (and pups born in the experimental setup).

^†^The colony includes only pups which were born in the experimental setup (though they can already be adults in late recordings; refer to dates of birth in Table 2 for details).

^‡^When only two bats were housed in a colony chamber, only one of two compartments (the ‘roost’) was used (see Fig. 1a).

^§^Recording channel switched from 9 to 10 on Nov. 1st 2012.

^||^Recording channel switched from 9 to 8 on Jan. 22nd 2013.

**Table 2:** Description of recorded subjects.

**Bat ID**	**Mother ID**	**Date of birth**	**Sex**	**Treatments**	**#Rec. Emitted**	**#Rec. Involved**
101	NA	Adult	M	1	197	903
102	NA	Adult	F	2	307	525
103	104	2012-06-28	M	3;7	62	152
104	NA	Adult	F	3	0	50
105	106	2012-05-19	F	4;7	73	112
106	NA	Adult	F	4	0	30
107	NA	Adult	F	1	156	274
108	107	2012-06-05	NA	1;5	190	247
109	NA	Adult	F	2	1329	2103
110	NA	Adult	F	2	1058	1791
111	110	2012-05-09	F	2;6;9	1341	2393
112	NA	Adult	F	1	271	582
113	112	2012-06-10	NA	1	97	103
114	NA	Adult	F	1	180	478
115	114	2012-05-20	F	1;5	230	359
116	NA	Adult	M	2	159	327
118	NA	Adult	F	1	62	190
119	NA	Adult	F	2	786	1249
120	119	2012-06-05	NA	2;6;9	659	1443
201	NA	Adult	F	10	634	2402
202	202	2012-09-25	NA	10	148	330
203	NA	Adult	F	10	579	2847
204	NA	Adult	F	10	1086	2949
205	NA	Adult	F	10	426	1779
207	209	2012-10-02	M	11;17;18;20	1245	15277
208	204	2012-09-22	M	10;16;19;20	779	13947
209	NA	Adult	F	11	0	92
210	212	2012-09-30	NA	12;17;18	1493	5132
211	205	2012-09-22	F	10;16;19;20	2225	8264
212	NA	Adult	F	12	0	3
213	NA	Adult	F	9	1239	2734
214	213	2012-09-12	NA	9	91	272
215	217	2012-10-04	F	13;17;19;20	6150	15945
216	226	2012-09-12	F	9;16;18;20	2524	7843
217	NA	Adult	F	13	0	113
218	NA	Adult	M	10	67	4029
220	224	2012-10-14	NA	14;17;19	1426	9270
221	228	2012-09-26	M	9;16;18;20	765	11119
222	225	2012-09-25	NA	10	102	245
223	NA	Adult	F	10	92	213
224	NA	Adult	F	14	0	72
225	NA	Adult	F	10	468	1614
226	NA	Adult	F	9	2310	4062
228	NA	Adult	F	9	1653	4238
230	232	2012-10-14	NA	15;17;18	3269	7946
231	203	2012-09-22	M	10;16;19	3051	11206
232	NA	Adult	F	15	0	41
233	NA	Adult	M	9	168	7737
0	Unknown	7858	21073			
Minus-sign (‘−’)	Unknown whether the individual is the emitter or the addressee	44075	44710			
*Bat ID* is used in the *emitter* and *addressee* columns in the annotations file. For pups which were born in the experimental setup the *Mother ID* and *Date of birth* are given; the others were captured in the wild as adults. *Treatments* as specified in Table 1. #*Rec. Emitted* is the number of recorded files containing vocalizations emitted by each bat (a recorded file may contain several vocalizations). *#Rec. Involved* is the number of recorded files containing vocalization emitted by or directed at the bat. *NA*—Not Available. *Minus-sign*ed emitter and/or addressee (e.g., −201) designates a common situation in which the pair of the interacting bats were recognized but their roles are in doubt (i.e., the call could be emitted by either bat).						

**Table 3:** Annotation details.

**Column**	**Description**	
1	File ID	File Identifier with properties detailed in FileInfo.csv (Data Citation 1).
2	Emitter	Bat ID of the emitter of the vocalizations. Negative value: the specified ID is of either the emitter or the addressee.
3	Addressee	Bat ID of the addressee of the vocalizations.Negative value: the specified ID is of either the emitter or the addressee.
4	Context	The context of the vocalizations as specified in Table 4.
5	Emitter pre-vocalization action	The action performed by the emitter of the vocalization before the start of the vocal interaction.
6	Addressee pre-vocalization action	The action performed by the addressee of the vocalization before the start of the vocal interaction.
7	Emitter post-vocalization action	The action performed by the emitter of the vocalization after the end of the vocal interaction.
8	Addressee post-vocalization action	The action performed by the addressee of the vocalization after the end of the vocal interaction.
9	Start sample	The annotation refers to the section beginning at this sample in the file (File ID, WAV format)
10	End sample	The annotation refers to the section which ends at this sample in the file.
Descriptions of each column of the annotation file.		

**Table 4:** Annotated contexts and behaviours.

**ID**	**Context/Behaviour**	**Description**	**# Rec.**
*Context*			
0	Unknown	Unknown context	640
1	Separation	Emitted (rarely) by adults when separated from the group.	504
2	Biting	Emitted by a bat after being bitten by another.	1788
3	Feeding	The interaction involves food.	6683
4	Fighting	The interaction involves intense aggressive physical contact.	7963
5	Grooming	The interaction involves one bat grooming another.	383
6	Isolation	Emitted by young pups.	5714
7	Kissing	The interaction involves one bat licking another's mouth.	362
8	Landing	The interaction involves one bat landing on top of another.	16
9	Mating protest	Emitted by a female protesting a mating attempt.	2338
10	Threat-like	The interaction involves contactless aggressive displays.	1065
11	General	Unspecified context. The interacting bats are usually 10–20 cm apart (in other interactions the bats are usually closer).	29627
12	Sleeping	The interaction occurs in the sleep cluster.	33997
*Pre-vocalization action*			
0	Unknown	Unknown action	13553*
1	Fly in	The bat approached the interaction location flying.	3909*
2	Present	The bat was present in the interaction location before the interaction started.	158164*
3	Crawl in	The bat approached the interaction location crawling.	6534*
*Post-vocalization action*			
0	Unknown	Unknown action	13437*
1	Cower	The bat cowered, partially covering its head with the wings.	77*
2	Fly away	The bat left the interaction location flying.	6745*
3	Stay	The bat stayed at the interaction location after the interaction ended.	155485*
4	Crawl away	The bat left the interaction location crawling.	6416*
Descriptions of the contexts and behaviours appearing in the annotation file, and the number of recordings obtained for each category.			

*Counted for both the emitter and addressee in each interaction.
